# Integrating ART adherence support technologies in the care of pregnant and postpartum people with HIV: a qualitative study

**DOI:** 10.1186/s43058-022-00331-0

**Published:** 2022-08-02

**Authors:** Sara Rendell, Harald Schmidt, Rebecca Neergaard, Hervette Nkwihoreze, Zoe Barbati, William R. Short, Aadia I. Rana, Anandi N. Sheth, Rachel K. Scott, Sonia Sethi, Florence M. Momplaisir

**Affiliations:** 1 Perelman School of Medicine, University of Pennsylvania, 3400 Civic Center Blvd, Philadelphia, PA 19104 USA; 2 Department of Medical Ethics and Health Policy, University of Pennsylvania Perelman School of Medicine, Philadelphia, PA USA; 3 Leonard Davis Institute of Health Economics, University of Pennsylvania, Philadelphia, PA USA; 4 Department of Family Medicine and Community Health, Perelman School of Medicine, Philadelphia, PA USA; 5 Department of Medicine, Division of Infectious Diseases, University of Pennsylvania, Perelman School of Medicine, Philadelphia, PA USA; 6 Department of Medicine, Division of Infectious Diseases, University of Alabama at Birmingham School of Medicine, Birmingham, AL USA; 7 Infectious Diseases Program, Grady Health System, Atlanta, GA USA; 8 Department of Medicine, Division of Infectious Diseases, Emory University School of Medicine, Atlanta, GA USA; 9 MedStar Health Research Institute and Washington Hospital Center, Washington, DC USA; 10 Department of Medicine, Rush University Medical Center, Chicago, IL USA

**Keywords:** Adherence, HIV care continuum, Pregnancy, Postpartum period, Anti-retroviral agents (ARV), Implementation science

## Abstract

**Background:**

We have a limited understanding on how to best integrate technologies to support antiretroviral therapy (ART) adherence in routine HIV care.

**Methods:**

We conducted semi-structured interviews with multidisciplinary providers caring for pregnant and postpartum people with HIV and asked providers about their perspectives on utilizing adherence support technologies such as text messages, video check-ins with providers or automated with facial recognition for directly-observed-therapy, signaling pill bottle, and signaling pill to support ART adherence. Each approach generated an adherence report. The interview instrument was guided by the Consolidated Framework for Implementation Research and included questions on the implementation climate, barriers, and facilitators to the clinical integration of the adherence approach and strategies that could be used to maximize this integration. The order of adherence support technologies was randomized to minimize bias. We used a modified grounded theory to develop the coding structure and two coders applied the codebook to the transcripts after establishing strong inter-rater reliability with 20% of interviews (kappa = 0.82).

**Results:**

Between March and December 2020, we conducted 26 in-depth, semi-structured interviews with providers who weighed several factors when considering each approach, including the approach’s effect on patient-provider interaction in and outside of the clinic visit, timing for and duration of the approach’s utility, threat of disclosing status, and added burden to providers (e.g., needing to act on generated information) or to patients (e.g., needing to hide the signaling pills, responding to text messages). Providers’ most preferred approach was text-messages, and the least preferred was the signaling pill. Barriers to acceptability varied by approach and included perceived surveillance, violation of privacy, added time demand for providers, potential inaccuracy of the adherence data generated, and negative impact on the patient-provider relationship, particularly if the approach was perceived as coercive. Payers anticipated regulatory hurdles with unfamiliar approaches, particularly the signaling pill and signaling pill bottle. Facilitators included strengthened therapeutic alliance, predictable reminder mechanisms, and options for customization according to patient preference.

**Conclusions:**

Our study elucidates barriers and facilitators to integrating technology-based adherence support approaches in clinical care to support adherence of pregnant and postpartum people with HIV.

Contributions to the literature

- Health care providers’ perspectives are crucial to inform the successful implementation of evidence-based approaches. Technology-based approaches that support adherence to antiretroviral therapy (ART) offer objective measures of ART adherence during pregnancy and the postpartum period, when many people experience HIV treatment interruption.
- Providers identified numerous barriers, including the threat of compromising the patient-provider relationship, and facilitators, including accessibility to patients and opportunities for more contact with the multidisciplinary team.
- These findings reveal barriers and facilitators of each approach and clarify how, when, and why providers might utilize each approach to improve the care of pregnant and postpartum people with HIV.

## Background

Antiretroviral therapy (ART) is an evidence-based practice that halts progression of maternal HIV and reduces the risk of perinatal transmission [1]. Recent estimates indicate that on average, 73.5% of pregnant people are adherent to ART, with adherence as low as 53% in the postpartum period [2]. Adherence is often suboptimal, especially in the postpartum period due to numerous factors including deprioritization of postpartum health relative to prenatal health, poor social support, increased financial stress, immediate time demands of caring for the newborn, and occasionally loss of access to benefits and entitlements available during pregnancy [3–5]. Delays in identifying lapses in adherence aggregate health consequences of lapses in adherence [4]. Successful implementation of interventions aiming at supporting ART adherence for people with HIV in the perinatal period is lacking [4]. Technology-based adherence support approaches are promising for addressing these gaps because they use automated mechanisms to increase accuracy of adherence patterns and thus can facilitate provider-patient discussions around ART adherence. In addition, they can be combined with other effective strategies, including peer support and case management, to identify and address barriers to ART adherence. Examples of technology-based adherence support approaches include text message reminders, video calls with providers, automated video check-ins, electronic pill bottles that send signals when containers are opened, and pills with embedded sensors [6–10]. There is increasing evidence that technology-based interventions change health-related behaviors and improve retention in care, with studies demonstrating improved ART adherence for people enrolled text messaging programs and ongoing trials of video based interventions for women with HIV [11, 12] These approaches are attractive as potentially low-effort opportunities for providers to partner with patients to bolster adherence during periods when it is deprioritized relative to other tasks and obligations. Barriers and facilitators to their integration in clinical care have not been well described and are needed to enable provider teams to support patients struggling with ART adherence. We used the Consolidated Framework for Implementation Research (CFIR) to elucidate perspectives of HIV providers and information about how each approach is situated within existing workflows and systems [13, 14] that can inform the implementation of adherence support approaches in clinical practice.

Provider perspectives are crucial to the successful implementation of adherence support approaches as provider buy-in is needed for the successful uptake of these approaches [15–17]. To date, few studies have elicited the attitudes of providers who care for pregnant or postpartum people with HIV regarding technology-based adherence approaches, even though these providers decide about the implementation of various adherence supports for their patients [18–20]. The current study aimed to address this gap in the literature and reduce the gap between research and practice by assessing provider perspectives on the pros and cons of each approach and on how these approaches can be integrated in clinical care.

## Methods

We conducted semi-structured interviews with multi-disciplinary providers as part of an ongoing multi-site study testing a peer-led behavioral intervention to improve adherence and retention in care for pregnant and postpartum people with HIV [21]. They included members of a comprehensive care team providing obstetric and HIV care and services to this patient population (advanced practice providers (APP), registered nurses (RN), perinatal case managers, and HIV-specialized physicians, including OB/GYNs). For this study, the term “providers” refers to the range of healthcare professionals providing care and services to pregnant/postpartum people with HIV, not only physicians. We use gender-neutral language in this manuscript in keeping with the CDC’s Health Equity Guiding Principles for Inclusive Communication [22]. In keeping with CFIR which considers a diversity of perspectives for the implementation of interventions, we also interviewed health payers (insurers) with a senior role in benefit design (patient interviews are ongoing as of this writing). This study was conducted in four sites that the U.S. Health and Human Services identified as priority jurisdictions for Ending the HIV Epidemic because of elevated incidence, e.g., where more than 50 percent of new HIV diagnoses occurred in 2017: Philadelphia, PA, Washington DC, Atlanta, GA and Birmingham, AL [23]. We recruited providers from clinics funded by the Health Resources and Services Administration’s Ryan White HIV*/*AIDS Program (RWHAP). Nearly three quarters of RWHAP clients are from racial/ethnic minority populations, and nearly two thirds of RWHAP clients are living at or below 100% of the federal poverty line [24].

The University of Pennsylvania Institutional Review Board approved the study (protocol number: 842757), and written informed consent was obtained from all study participants*.* To avoid conflicts with clinical responsibilities, interviews were scheduled according to each participant’s availability over a ten-month period, from March through December of 2020.

### Recruitment

We identified providers and payers through purposive sampling to target a variety of perspectives at each site. Clinical directors identified most experienced staff members who were then invited to participate in the study by a research specialist from the Mixed Methods Research Lab (MMRL) at the University of Pennsylvania. Participants were also asked to recommend colleagues of good fit for the project, who were then contacted for an interview.

Recruitment stopped after we met target numbers for each category, determined based on the maximum number of staff in each role dedicated primarily to caring for pregnant and postpartum people with HIV and if saturation of themes based on barriers and facilitators mentioned across each approach was met. We targeted a total of 13 providers (8 physicians, 1 APP, 4 RN, 9 case managers) and two payers with senior role in benefit design), with a goal of a minimum of 25 interviews.

### Study instrument

The study collaborators designed a survey instrument in collaboration with an MMRL specialist drawing on the Structural Vulnerability Assessment Tool which elucidates the pathways through which specific local hierarchies and broader sets of power relationships influence health [25] and the CFIR [13, 14]. Within the CFIR, key domains include (1) intervention characteristics (i.e., characteristics of each adherence support approach); (2) outer setting (i.e., the economic, political, and social context within which an organization exists); (3) inner setting (i.e., the structural and cultural climate through which an implementation process proceeds); (4) characteristics of staff involved in implementation; and (5) the implementation process [13].

Specialists in instrument design, interviewing, and qualitative analysis at the MMRL then piloted the survey instrument with participants and made minor adjustments to the instrument based on relevance and clarity of each question. The final interview instrument included 36 questions grouped into several themes relevant to the implementation climate including narratives for non-adherence, perceptions about monitoring and tracking adherence, and barriers and facilitators regarding the five adherence support approaches (namely, text message reminders, video check-ins with providers for directly observed therapy, automated video check-in with facial and pill recognition for directly observed therapy, a signaling pill bottle, or a signaling pill), accompanied by a one-page visualization of each support approach (Table 1). The one-page visualization offered to providers detailed how each approach enabled a given patient’s adherence to be recorded, either manually as with video check-ins with providers or automatically (all other options).

**Table 1:** Adherence support approaches

Adherence approach	Description
**Signaling pill**	Smartphone app reminds patients when it is time to take their pill. Each pill is fitted with a sensor and when it reaches the stomach, the sensor sends a signal to a computer system. The computer system records whether and when the pill was taken, and adherence records can be automatically shared with others.
**Signaling pill bottle**	Pill bottle flashes light when it is time for the patient to take their pill. When the cap is removed, the pill bottle lid automatically sends a message to a computer system. The computer system records whether and when a pill bottle was opened, and adherence records can be automatically shared with others.
**Video check with provider**	A provider calls and observes patient taking their pill via smartphone or computer, using a video platform such as FaceTime or Skype. The provider records whether and when a pill was taken, and adherence records can be manually shared with others.
**Automated video check**	A computer program with facial and pill recognition ability calls the patient on their smartphone or computer. The computer program watches the patient take their pill and records whether and when a pill was taken. Adherence records can be automatically shared with others.
**Text messages**	Provider reminds patient it is time to take their pill via text message. The patient takes their pill and responds, reporting whether and when they took their medication. Adherence records can be shared with others.

The order in which approaches were presented and providers were asked about these approaches was randomized to minimize inadvertently influencing the conversation in favor of or against an approach. We left it to providers to determine the hypothetical frequency of use for each approach based on their clinic’s capacity to enable further characterization of the implementation climate. After completing interviews, providers were invited to participate in a brief demographic survey that asked about provider role, self-identified gender, self-identified race/ethnicity, age and years of experience in HIV care, years of experience working in RWHAP clinics, and years of experience caring for peripartum people.

### Data analysis

All interviews were conducted in English and were carried out by an MMRL specialist with whom interviewees did not have a preexisting relationship. Interviews were audio recorded and transcribed in their entirety, and verbatim transcripts were coded. Mean interview duration was 36 min (range 22–53 min). NVivo 12 Plus was used for coding of interview transcripts [26]. We utilized a modified grounded theory approach, which is inductive in nature, to allow themes of importance to providers interviewed to be represented in the data and to inform the development of our codebook. Study team members reviewed and discussed the codebook at coding meetings and refined it using an iterative process that included coding to question, theme generation, and subsequent coding to theme. Any new themes emerging during coding prompted changes to the codebook. Two coders from the MMRL then applied the resulting codebook and established strong inter-rater reliability with 20% of interviews (kappa = 0.82). The remaining interviews were divided between reviewers and coded independently. A table summarizing provider perspectives (Table 2) was generated from coded interviews by identifying where codes for each adherence support measure overlapped with reflections on them, including manual counts of frequency of appearance of each idea in interview transcripts.

**Table 2:** Overview of provider perceptions of adherence support approaches

		Effect on patient-provider relationship	Effect on provider workflow	Threat of status disclosure	Access and ability	Suggestions for customization
**Text message**	Facilitators	Patient can form a relationship with provider they text (1)^a^	Providers can automate (1) and access adherence data (1)	Offers more privacy than call options (2)	Most patients able to text (9)	Personalize the text message (2) Combine with the signaling pill box (1) Use to help patients start a routine and then stop (3)
	Barriers	Fear of becoming a nuisance to patients after repeated messages (1)	Burdens providers to text many patients at all hours (5)	Risk of disclosure to others who have access to patient’s phone (4)	Some patients lack a phone or have limited texting ability (2)	
**Video check with provider**	Facilitators	Patient can develop a close relationship with provider (12)	NA	NA	NA	Valuable at particular times in pregnancy (2) Use for limited period while patient is developing a routine (3) Assure the person calling has a relationship with the patient (1) Train patients to use the technology (1)
	Barriers	Patient may feel uncomfortable being watched (1)	A lot of work for provider to call all patients every day to watch them take their pills (9)	Possibility patient is around others who are not aware of HIV status at time of video call (5)	Patient must have and understand technology necessary for video calls (4)	
**Automated video check**	Facilitators	NA	Requires less labor and money, but gives the same amount of adherence data (7)	NA	NA	Tailor interaction to the patient and change it regularly to retain engagement (1) Calls start with a provider and transition to automated over time (1)
	Barriers	Lack of opportunity to connect with provider (4)	NA	Possibility patient is around others who are not aware of HIV status at time of video call (1)	Patient must have and understand technology necessary for video calls (4)	
**Signaling pill bottle**	Facilitators	Patient can be proud to show adherence record to provider (1)	Little work for providers to do with this intervention (2)	NA	Method of getting and taking pills does not change (1)	Have someone call the patient if pill cap is not opened (2) Add a second reminder if pill cap is not opened (1) Send a text message along with the reminder light (2)
	Barriers	Signaling distrust by tracking adherence (1)	Someone must organize data (1)	Flashing light can attract unwanted attention to medication (9)	NA	
**Signaling pill**	Facilitators	Patient gets additional support without having to reach out for it (1)	Tech does the work of checking up on patients for the provider (2)	NA	NA	Only useful for a short period of time (1)
	Barriers	signaling distrust by tracking adherence (2)	someone must monitor adherence data (1)	concerns about tracking device being tied to HIV status (2)	need a smart phone and comfort with technology (2)	

^a^Numbers in parentheses represent number of providers who explicitly mentioned each factor; NA signifies not applicable as none were mentioned

## Results

Provider characteristics are presented in Table 3. Providers were primarily female (87%), and from diverse disciplines a majority of whom (78.3%) had six or more years of experience in HIV care.

**Table 3:** Interview participant characteristics

	Number	Percentage
**Role**		
Physician	8	30.8%
Nurse practitioner	1	3.8%
Nurse	4^a^	15.4%
Case manager	10	38.5%
Insurer (payer)	3^a^	11.5%
Total	26	
**Years of experience in HIV care**		
0–5	5	21.7%
6–10	4	17.4%
11 or more	14	60.9%
Total	23^a^	
**Experience in Ryan White clinics (years)**		
0–5	4	17.4%
6–10	6	26.1%
11 or more	10	43.5%
NA	3	13.0%
Total	23^a^	
**Experience with peripartum patients (years)**		
0–5	6	26.1%
6–10	6	26.1%
11 or more	10	43.5%
NA	1	4.3%
Total	23^a^	
**Self-identified gender**		
Male	2	8.7%
Female	20	86.9%
Other	1	4.3%
Total	23^a^	
**Self-identified race/ethnicity**		
Asian	2	8.7%
Black or African American	8	34.8%
Hispanic or Latinx	1	4.3%
White	11	47.8%
NA	1	4.3%
Total	23^a^	
**Age**		
20–39	7	30.4%
40–59	13	56.5%
60+	2	8.7%
NA	1	4.3%
Total	23^a^	

^a^There were 3 insurers interviewed for the study; however, 2 of the 3 insurers did not complete the demographic survey. Similarly, 4 RNs were interviewed; however, 1 RN did not complete the demographic survey

### Intervention characteristics

Providers explicitly prioritized approaches that they felt would enhance patients’ overall wellness and promote patient-provider trust. A majority of providers expressed familiarity with text messaging, a plurality of providers expressed familiarity with an automated or live-provider video check, and a few providers implied familiarity with the signaling pill or signaling pill bottle. Text messaging was the most popular approach because it was familiar to providers, easily accessible to patients, and could enhance patient-provider relationships. The text message approach was also perceived as less invasive compared with other approaches. There were greater concerns about privacy and surveillance for the signaling pill, signaling pill bottle, video check with provider, and automated video check.

### Outer setting for adherence support approaches

Providers situated adherence among many challenges their patients navigate, including housing instability, food insecurity, and legal difficulties. “I have not found adherence itself to be the major problem, but more the steps before it. […]” (Physician). One payer with prior experience as a HIV physician described housing as the “fifth vital sign.” Discrimination and hardship based on race, immigration status, and socioeconomic status were cited as consistent contributors to nonadherence. Payers understood adherence as subject to rapid changes: “Somebody can be completely 100% adherent for six months and then things can happen in their lives that drop off”(Payer). Case managers observed that adherence declines when patients lack the basic security of stable shelter and ability to pay bills and feed oneself and one’s family: “It’s usually not a medication access thing. It's, ‘Oh, I take the medication, and I need to eat with it, but I didn't have any food, so I missed the med because I didn't have any food’” (Case Manager). For providers, connection to care was inextricable from medication adherence. Actions taken to identify and ameliorate the upstream, outer context causes for non-adherence were understood by providers to be intrinsic to, rather than separate from, their clinical responsibilities. Providers tended to view “human connection” as integral to identifying such factors and to developing collaborative plans to address them. Providers consistently identified the postpartum period as particularly challenging for retention in care and ART adherence, citing comparatively fewer resources available than during pregnancy, as one physician put it, “there is loss of insurance, pregnancy [coverage], and sometimes their source of payment for the medication changes.” Dominant external setting themes in provider explanations for declining adherence postpartum included increased financial, cognitive and physical demands in the setting of sometimes loosing access to financial supports available during pregnancy; direct competition between care for self and care for newborn; declining risk of avoiding perinatal transmission; and postpartum depression. Providers did not view technology-based approaches as a solution to addressing social determinants but as one of many tools that could be used to better support patients’ ART adherence in the postpartum period.

### Inner setting for adherence support approaches

Providers weighed the effect of each approach on patient-provider interactions in and outside of the clinic visit and the possible added burden to providers or clinic staff, including having to act on information once it was known. Providers also thought that added burdens could extend to patients; for example, patients might need to store the signaling pills in a new location as the signal emitted might attract unwanted attention for individuals who have not disclosed their status. Patients might also feel that they are expected to respond to text messages. Several providers described that having a low number of patients who are pregnant or postpartum, compared to the larger clinic volume, could serve as a facilitator to the integration of a new technology for this population, by reducing the overall time burden required to respond to output. Others remarked that the data provided by these approaches could easily be integrated on an existing electronic health record (EHR) and to other EHR-based initiatives they had adopted to assess practice-wide adherence metrics. Providers across sites consistently described human effort and burden (time and tasks) as key factors they would weigh in when considering which adherence support approach to select.

### Implementation process

The most consistently cited facilitators were enhancing patient-provider relationship, predictable reminder mechanisms, and options for customization based on patient preference. Payers anticipated regulatory hurdles with unfamiliar approaches, particularly the signaling pill and signaling pill bottle. Below, we discuss barriers and facilitators to each approach in detail and strategies that can be used to integrate use of the approach in clinical care. Table 2 provides an overview of associations providers made between adherence support approaches and factors they deemed important, detailed results from Table 2 are summarized by approach as follows.

### Text messages

Providers saw text reminders as an opportunity to develop better and more frequent interactions with patients. They believed text message reminders and report could be helpful for most patients, as most have text-capable devices, check them frequently, and know how to text. Providers presumed younger patients would text more often and more seamlessly utilize the intervention. Many providers had successfully used text messages to contact their patients in the past.

Barriers included the ease of ignoring a text message or responding dishonestly which were seen as a diminishing return for investment. The frequency of text messages was also concern. One physician shared, “my general experience is that after two-to-four weeks, they began to ignore [text reminders]. You never want to get to that place that the patient is now avoiding the interaction.” Providers expressed concerns about HIV status disclosure through a text message, especially if a patient shared a phone. Several case managers described how even a discrete message could become “a potential outing for that patient” (Case Manager). Providers suggested that customizing reminder texts could resolve issues around disclosure, potentially facilitating use of the approach.

### Video check with providers

Facilitators to this approach included a sense that video checks could improve the therapeutic alliance, as a “social approach” seen to enable “human connection,” a factor thought to mitigate outer context barriers providers emphasized. Providers framed the video check as best for patients who enjoy person-to-person contact. Providers suggested newly diagnosed patients, patients switching medications, and postpartum patients for this approach. Overall, providers conceptualized video calls as a temporary tool for establishing or strengthening an adherence routine.

Barriers to this intervention included high demand on providers, requirement of video-calling capable devices, and the possibility of becoming cumbersome to patients: “It would take a lot of time and a lot of resources to make sure that patients have access to a smartphone and can Skype or FaceTime” (Case Manager). Providers feared a video call would make patients feel invaded: “With our population with the stigma and all of it, I don't think that'll work because [if] somebody is watching [a patient take a pill], the [patient] probably will feel violated” (Case Manager).

### Automated video check

Providers were less enthusiastic about the automated video check, and few thought it would appeal to patients. Barriers included perceiving this option as a less personalized approach and one whose facial recognition technology risked patient discomfort. Generally, providers felt the automated call incurred the same effort of a live video call without the benefits of human connection with a provider.

“This method doesn’t offer any of the support that the video potentially could. The video that we usually do is like a quick check in. “How are you feeling? Are you ready to take your pills today? Great, let’s take it. Oh, you did such a good job.” There’s a positive reinforcement as opposed to a video just recording.” (Physician)

However, some thought it might help non-adherent patients who found personal contact burdensome. Providers viewed the automated video call as less resource-intensive for providers and less intrusive for patients and saw it as a possible method for stepping down from personal contacts.

### Signaling pill bottle

Facilitators to this approach included an appreciation of the novelty of this visual reminder (e.g., remarking that a blinking pill bottle was an unheard of and exciting way to help patients remember whether they had taken their medications on a given day) and a sense that it would not disrupt routines for picking up and taking pills, even though it could change how patients store pills.

However, providers remarked that flashing pill bottles are not discreet for those trying to keep their status private who, for example, conceal their pills in a vitamin bottle. “Anything that would draw attention to the medication would be something they would want to avoid” (Physician). In addition to disclosure concerns, providers noted that the signaling pill bottle could be ineffective for patients who do not store their pills in the original prescription bottle or in a visible location (for example, keeping pills in a pill organizer or drawer). One physician worried the signaling bottle could communicate an “assumption that you don't trust the patient being able to take their meds without being monitored.” The most common concern among providers was that the data from the signaling pill bottle could be misleading if a patient opened the bottle but did not take their pill.

### Signaling pill

Many providers thought patients would dislike swallowing a sensor due to feelings of being watched or having their privacy invaded. Additionally, some providers worried that relying on the signaling pill rather than patient report to assess adherence could threaten the patient-provider relationship. “In some ways, it’s signaling a lack of trust to the patient” (Case Manager). Providers believed the signaling pill would be ideal for patients who were chronically non-adherent and dishonest in reporting missed pills, though they speculated those patients would not accept the approach. Facilitators included an appreciation of the accuracy of information from the signaling pill, even if few expressed willingness to use it.

### Mixed reactions to detailed record keeping on medication adherence

Overall, providers differentiated supporting from verifying adherence, despite the potential for each approach to combine both functions. When asked whether they would like a detailed report of whether and when their patients take their medications, providers offered mixed responses. A plurality of providers (*N*=12/26) speculated that information would be “*a nice tool*” or could enable them to locate specific causes for missed doses. One provider felt positively about this option but added, “*I’d definitely question how that information is collected and the validity and the [re]liability*[…]” (RN). Others thought a detailed report would contribute little to their preexisting clinical practice. Overall, providers emphasized that having data does not lead directly to having the resources and capacity to address the problem(s) the data reveal.

## Discussion

Provider perspectives on implementation of adherence support technologies for pregnant and postpartum people with HIV included an explicit focus on how to best care for each patient, noting heterogeneity in social, economic, and structural vulnerabilities that could influence adoption of each approach. While providers perceived opportunities for each of the approaches, they consistently expressed concern about possible detrimental effects: the most frequently cited barrier was the threat of compromising the patient-provider relationship and the most consistently mentioned facilitators were perceived accessibility to patients and the opportunity to forge more contact with the multidisciplinary team. There are a few studies of adherence support approaches that directly assess patient experiences (namely, SMS messaging and pill bottle caps with embedded sensors transmitting signals with bottle opening) [27–29]. An exploratory study of pill bottle caps with embedded sensors to monitor adherence among 12 African American women living with HIV revealed 10 out of 12 participants consistently used the system during the 30-day study period and that subjective adherence was lower than objectively recorded ART adherence [27]. Interviews of women using text message-based approaches to adherence revealed acceptability among patients was high and main concerns were risk of unintended HIV status disclosure [28]. These findings mirror provider concerns about confidentiality in the current study. However, focus group discussions among participants of an RCT assessing text-message based adherence support revealed that a majority of participants opted to receive text messages that overtly refer to HIV status, suggesting that patients who experience relatively low risk of status disclosure by text message may prefer more direct communication regarding HIV [29].

Provider effort and added burdens on patients, and care team members were strong considerations informing the feasibility of integrating each approach within clinical care. Past experiences with the approach positively influenced acceptability to providers, which partially explains why text messaging was the preferred approach. Additionally, familiarity with an analogous approach positively influenced feasibility as providers considered scenarios by which the data generated could be integrated in the EHR. These results indicate that adherence support approaches can be effective tools to enhance ART adherence if they are customized to the needs of patients; adapted to clinic resources, and strategies to address barriers to their integration in clinical care are used.

In our study, providers emphasized outer setting factors influencing adherence, including housing instability and discrimination. Such an emphasis is consistent with scientific evidence that has established the significant influence of such factors on women’s HIV self-management [30–32]. Automated systems cannot address structural barriers by themselves but do present opportunities for task-shifting which could free time providers spend on adherence assessment during patient encounters to be instead devoted to addressing barriers to ART adherence [33]. However, in the current study, providers tended to more frequently anticipate that these approaches would increase workload, unless the approach was integrated with an existing data management system or adapted to address patient preferences, including frequency of contact and preservation of privacy. One recent study assessing provider perceptions of a mobile messaging intervention designed to encourage patients to remain in care found that health care providers tended to balance considerations of practicality and added workload with potential to improve patient-provider relations and weighed the latter more heavily [33]. Provider weighting was again consistent with scientific evidence revealing that positive relationships with providers and clinic staff facilitate retention in care for patients with HIV [34]. Overall, the findings of this study suggest that successful implementation of existing adherence supports requires prioritization of efforts that address broader structural barriers this patient population faces, and therefore it is important that these upstream factors be considered in any efforts to implement adherence support technologies.

Our study has several limitations. Because it is descriptive in nature, it does not establish causal associations, describe the relative likelihood of providers to use a specific approach, or allow subgroup analysis to compare perspectives among providsers. Though the order in which adherence supports were discussed was randomized, the ordering may nonetheless have inadvertently influenced perspectives about their relative value. The interviews were conducted during the first few months of the COVID-19 pandemic, and provider perspectives may have reflected pandemic-related changes in clinical practice since the time of the interviews. This study included the perspectives diverse providers across four cities where HIV infection remains disproportionally high among Black and Hispanic/Latina patients [23]. Our findings contribute to the literature by describing barriers and facilitators of each technology-based approach and clarify how, when, and why each approach might be utilized by a provider in clinical practices to improve the care of pregnant and postpartum people with HIV.

## Conclusions

The findings of this study reveal barriers and facilitators of each technology-based approach and clarify how, when, and why each approach might be utilized by a provider in clinical practices to improve the care of pregnant and postpartum people with HIV. Although providers readily identified practical utility of each approach, they cited numerous barriers to use, including the threat of compromising the patient-provider relationship, and facilitators, including perceived accessibility to patients and the opportunity to forge more contact with the multidisciplinary team. The successful implementation of adherence support approaches requires emphasis on the provider-patient relationship, as well as concomitant structural, interpersonal, and intrapersonal difficulties patients balance with adherence to ART. Future research should integrate provider and patient perspectives on the proliferating approaches to HIV adherence support, provide quantitative assessment of factors that inform provider receptivity to various approaches, and broaden to include complementary approaches to improving outcomes for chronic conditions that often co-occur for people with HIV [35].

## Data Availability

The datasets used and/or analyzed during the current study are available from the corresponding author on reasonable request.

## Abbreviations

APP: Advanced practice provider
ART: Antiretroviral therapy
CFIR: Consolidated Framework for Implementation Research
HIV: Human immunodeficiency virus
MMRL: Mixed Methods Research Lab
PrEP: Pre-exposure prophylaxis
RN: Registered nurse
RWHAP: Ryan White HIV/AIDS Program
